# Digital Twins in Agriculture and Forestry: A Review

**DOI:** 10.3390/s24103117

**Published:** 2024-05-14

**Authors:** Aristotelis C. Tagarakis, Lefteris Benos, George Kyriakarakos, Simon Pearson, Claus Grøn Sørensen, Dionysis Bochtis

**Affiliations:** 1 Institute for Bio-Economy and Agri-Technology (IBO), Centre for Research and Technology-Hellas (CERTH), 6th km Charilaou-Thermi Rd., 57001 Thessaloniki, Greece; e.benos@certh.gr (L.B.);; 2 farmB Digital Agriculture S.A., Dekatis Evdomis (17th) Noemvriou 79, 55534 Thessaloniki, Greece; 3 Lincoln Institute for Agri-Food Technology (LIAT), University of Lincoln, Lincoln LN6 7TS, UK; 4 Department of Electrical and Computer Engineering, Aarhus University, 8000 Aarhus, Denmark

**Keywords:** open-field agriculture, livestock farming, forestry, digital twin definition, information and communication technology, level of integration, maturity level

## Abstract

Digital twins aim to optimize practices implemented in various sectors by bridging the gap between the physical and digital worlds. Focusing on open-field agriculture, livestock farming, and forestry and reviewing the current applications in these domains, this paper reveals the multifaceted roles of digital twins. Diverse key aspects are examined, including digital twin integration and maturity level, means of data acquisition, technological capabilities, and commonly used input and output features. Through the prism of four primary research questions, the state of the art of digital twins, the extent of their achieved integration, and an overview of the critical issues and potential advancements are provided in the landscape of the sectors under consideration. The paper concludes that in spite of the remarkable progress, there is a long way towards achieving full digital twin. Challenges still persist, while the key factor seems to be the integration of expert knowledge from different stakeholders. In light of the constraints identified in the review analysis, a new sector-specific definition for digital twins is also suggested to align with the distinctive characteristics of intricate biotic and abiotic systems. This research is anticipated to serve as a useful reference for stakeholders, enhancing awareness of the considerable benefits associated with digital twins and promoting a more systematic and comprehensive exploration of this transformative topic.

## 1. Introduction

### 1.1. Broader Perspective and Motivation

Agriculture has consistently been acknowledged as the sector tasked with addressing some of the most pressing challenges faced by humanity. These challenges encompass the growing global demand for food due to the substantial increase in the world’s population, adverse effects arising from climate change, natural resource depletion, and biodiversity decline [1]. In the face of urgent demands for increased agri-production effectiveness and concurrent reduction in environmental impact, the trajectory of agriculture production systems is rapidly evolving towards integrated precision agriculture practices. These practices indicatively include the non-uniform distribution of crop protection chemicals, soil nutrients, and irrigated water. “Precision agriculture”, a concept that dates to the mid-eighties, employs minimum quantities strategically targeted to specific locations [2,3]. Its primary objective is to enhance crop management by ensuring maximum productivity through accurate measurements informing targeted actions, thereby offering a comprehensive overview of farm processes. A notable subfield of interest is timely disease detection, as crop diseases pose a significant threat, causing a decline in both crop yield and quality. Several studies have addressed such applications, most of which elaborate machine learning algorithms trained to detect specific diseases in specific crops like anthracnose in walnut trees [4], tomato [5], and citrus [6], among others. Furthermore, early weed detection is vital for effective weed control through either mechanical intervention or herbicide application [7]. Within the broader domain of precision agriculture, there are also subdomains that specifically focus on precise management of water and soil resources, including variable rate application, soil health monitoring, and irrigation management.

Similarly to the need for precision in crop farming, “precision livestock farming” has surfaced as a comprehensive approach employing sensors and incorporating real-time monitoring to gather data for optimal management of livestock [8]. Precision livestock farming includes, among others, the automated identification of individual animals and the use of wearables for tracking and monitoring physiological parameters. Monitoring procedures are also used to identify any slight deviation from normal behavior (for the animal in question), facilitating early recognition of clinical disease symptoms [9]. Moreover, automated feeding systems have been developed and implemented, offering an interface to oversee the timing of feeding and the quantity of feed ingested [10].

Following the progress in agriculture, forestry operators worldwide have initiated the adoption of cutting-edge technologies to optimize forest management. This strategy is commonly referred to as “precision forestry”, following the corresponding pressure for improving effectiveness [11]. The set of technologies, methodologies, and knowledge employed may encompass a broad range of practices aiming at maximizing resource efficiency and minimizing environmental impact [12]. This innovative data-driven approach leverages advanced technologies to enhance informed decision-making related to forest inventory, tree species and age identification, tree health monitoring, and analysis of biodiversity patterns. Precision forestry also incorporates climate data and predictive models to evaluate the potential impact of climate change on forest ecosystems and formulate strategies for adaptation.

The common denominator among precision agriculture, livestock, and forestry lies in the shared pursuit of exploiting advanced technologies and data-driven approaches to optimize resource management and enhance the overall efficiency of both biotic and abiotic systems. Following the broader framework of the fourth industrial revolution, “Agriculture 4.0” [13,14] and “Forestry 4.0” [15,16] have emerged, constituting the evolution of respective precision concepts and ushering in a new era of “smart”, interconnected, automated, and sustainable practices. This paradigm shift transforms conventional production methods, driving corporations towards elevated productivity, minimized expenses, and heightened innovation. Within this digital evolution, Information and Communication Technology (ICT) integration plays a central role. ICT, an encompassing term, refers to various devices and applications facilitating data collection, exchange, and communication. In agricultural and forestry environments, ICT in an extended and broadened framework comprises a wide spectrum of technologies ranging from collar-mounted GPS devices [17], implantable temperature sensors [18], wireless sensor networks [19], and remote sensing [20] to agri-robotics [21], artificial intelligence [22], machine learning (ML) [23], cloud computing [24], and information management infrastructures [25].

Within this broader scope of ICT, Digital Twins (DTs) have emerged with the potential to revolutionize agriculture and forestry production systems by offering real-time insights, refining decision-making processes, and seamlessly integrating physical and digital entities. In essence, DTs function as a transformative factor boosting the intelligence, interconnectivity, and efficiency of physical systems, thus promoting growth and progress in diverse industries. DTs can be simply regarded as virtual representations of physical objects or systems formed by incorporating real-time data enabling, inter alia, monitoring, simulation, and analysis for the purpose of enhancing performance and supporting management of various processes. The practical implementation of DTs in agriculture and forestry experienced a delayed initiation in the previous decades. This can be attributed to some constraints of ICT, like limited computing power, challenges in data storage, lack of device connectivity due to poor coverage in most rural environments, and, most importantly, the information fragmentation among various technology solutions. However, driven by the advancement of digital transformation and the progress in a plethora of technologies, DTs seem to be moving from conceptualization to actualization at an accelerated pace.

### 1.2. Aim of the Present Study

Motivated by the remarkable progress in DT technology and its transformative potential, this review study focuses on the realm of DT applications in open-field agriculture, livestock farming, and forestry. It provides a thorough review of the relevant scientific literature concerning the development and application of DTs in the above sectors. To this end, the scholarly literature was meticulously screened to encompass a wide range of crucial aspects, including the maturity level of these applications and the level of DT integration, based on a rigorous research protocol. Owing to the specific nature of these ecosystems, the relevant published studies show discrepancies concerning the definition and application of DTs in these sectors. More specifically, they all commit to describing DTs in agricultural and forestry applications, but the level of digital representation differs, and, in many cases, it does not reach the DT level. This is attributed to the open nature of agricultural and forestry environments that lack discrete boundaries. Therefore, there is an urgent need to define the specifications and applications of DT in agricultural and forestry systems, a research gap that has been identified by the authors. Moreover, while existing literature often segregates discussions on the application of DT in these domains, the present review study provides a comprehensive analysis that aims at bridging them by implementing an integrated approach and presenting a cross-disciplinary perspective on how DTs can be leveraged seeking to uncover novel insights and potential cross-domain knowledge transfer. In addition, shared challenges, solutions, and future perspectives are identified and evaluated that may have been overlooked in singularly focused reviews.

### 1.3. Paper Outline

The subsequent sections of this paper are organized as follows: Section 2 provides a concise overview of the foundational concepts of the DT and associated technologies, aimed at enhancing the reader’s understanding of the subject of this study. In Section 3, the implemented methodology is detailed, encompassing the steps taken in the review process as well as the evaluation of both the technology readiness level and levels of integration of the reviewed DT applications. Section 4 presents the main findings in a tabular format, supplemented by bar and pie charts for visual clarity and understanding. Section 5 provides a discussion of the results based on the predefined primary research questions posed in the methodology section. Finally, Section 6 draws the main conclusions.

## 2. Brief Overview of the Digital Twin Concept and the Main Related Technologies

Historically, the foundational principles of the DT concept were pioneered by NASA in the 1960s through the development of a “living model” for the Apollo missions [26]. This model utilized a combination of physical simulations and digital elements to conduct an in-depth failure analysis for the ongoing assimilation of data. The more concretely shaped idea of DT, however, came from a conference organized by the Society of Manufacturing Engineering (SME) in Troy, Michigan, in October 2002 [27]. In this presentation, Michael Grieves introduced a conceptual model for managing the lifecycle of a product, thus marking the birth of the concept. The concept was latter named “*Mirrored Spaces’ Model*” [28], followed by “*Information Mirroring Model*” and “*Virtual Doppelganger*” and finally “*Digital Twin*” in 2014 [29,30]. Three fundamental components of the first conceptual model presented in [27] are: (a) physical space and its products (later defined as Physical Twin); (b) virtual or digital space and its products (later defined as Digital Twin); and (c) the connection between the two spaces (later defined as Physical Thread) [30]. These components persist even today, following Grieves’ foresight that the constrained, paper-based data collection methods prevalent at that time would be substituted by two-way digital data connections. These connections would facilitate the transfer of data from the physical entity to the virtual counterpart and, conversely, the transmission of information and processes from the virtual representation to the physical one.

The definition of DT, as the convergence between physical and virtual entities, has undergone several changes over time [31,32,33]. Indicatively, the CIRP Encyclopedia of Production Engineering [34] recently provided the following concise definition: “*A digital twin is a digital representation of an active unique product (real device, object, machine, service, or intangible asset) or unique product-service system (a system consisting of a product and a related service) that comprises its selected characteristics, properties, conditions, and behaviors by means of models, information, and data within a single or even across multiple life cycle phases*”. Additionally, as highlighted in [35]: “*Digital twins can also be linked to create twins of larger systems*”. DTs surpass conventional static product designs such as Computer-Aided Design (CAD) models, incorporating dynamic behavior. A virtual copy of a real-world asset is generated through continuous data transmission, enabling the simultaneous existence of the digital version alongside the physical one.

The core components of DTs encompass data acquisition, data modeling, and data application. To accomplish these tasks, according to [36], DTs usually leverage four key technologies that can work together to gather and store real-time data, extract valuable insights, and establish a digital representation of the physical entity:

- Internet of Things (IoT): It forms a vast network connecting entities such as things, people, or a combination of both. Within the IoT framework, various types of wireless sensor networks are used to collect data from real-world objects, enabling the creation of a digital replica for analysis, manipulation, and optimization.
- Cloud Computing: This technology equips DTs with computing and data storage capabilities. Consequently, DTs can store extensive data in the virtual “cloud” and access needed information from any location, effectively reducing computation time for complex systems and addressing challenges associated with large data storage.
- Artificial Intelligence (AI): AI aims to replicate the fundamentals of human intelligence, creating intelligent machines that mimic human intelligence. Key AI domains comprise robotics, computer vision, body language and vocal interaction, and ML [37]. DTs are enhanced by AI by serving as an advanced analytical tool, automatically analyzing data, offering insights, making predictions, and suggesting strategies to prevent potential issues.
- Extended Reality: Virtual, augmented, and mixed reality are subfields included within the broad term of extended reality. These technologies combine the physical and virtual worlds, expanding our experience of reality. In particular, virtual reality immerses users in entirely artificial environments, augmented reality overlays digital information to the real world, enhancing perception, while mixed reality integrates aspects of both.

DT, as an innovative technology, finds applications across diverse sectors, including the supply chain/logistics industry, healthcare, construction, manufacturing, 3D printing, aerospace, automotive, and power industries, as well as agriculture and forestry [36,38,39]. Interestingly, Gartner incorporated DT into its compilation of the top 10 technology trends for 2019 [35]. Finally, according to [40], the DT technology is anticipated to experience substantial growth in the coming years, projecting an increase from USD 10.1 billion in 2023 to USD 110.1 billion in 2028, with a Compound Annual Growth Rate (CAGR) of 61% throughout the forecast period.

Despite this promising outlook, DTs present considerable challenges. Implementation complexities and costs necessitate substantial investments in technology and infrastructure, with end users currently evaluating the associated economic benefits and long-term savings [41]. DTs rely heavily on massive amounts of diverse and high-quality data, posing challenges in data management, accuracy, and security, which require sophisticated solutions to ensure reliability [42]. Integrating DTs with existing systems is hindered by interoperability issues, particularly with legacy infrastructure. Additionally, the widespread adoption of DTs demands a transformation in workforce skills across the value chain, necessitating a dynamic environment for effective implementation [43,44]. Ethical considerations around data ownership, privacy, and bias further complicate adoption and require careful management [45].

## 3. Methods

### 3.1. Essential Systematic Review Steps

In the present review study, a sequence of six key steps was employed, mirroring relevant scholarly practices [46,47]:

1. Primary research questions (RQ) formulation:
  - RQ1: “How are DTs conceptualized and defined in agricultural and forestry systems?”;
  - RQ2: “What are the current applications of DTs in open-field agriculture, livestock, and forestry?”;
  - RQ3: “To what extent has DT integration been achieved?”;
  - RQ4: “What are the open questions, challenges, and future perspectives?”.
2. Research protocol establishment: A comprehensive research protocol was established, documenting the methodology for literature screening, data extraction, and analysis. This protocol received unanimous approval from all authors before initiating the literature search, aiming to minimize bias.
3. Literature search: The methodology for study selection is elaborated in Section 3.2, including the inclusive criteria, utilized electronic databases, and review stages adhering to PRISMA guidelines [48].
4. Data extraction: Specific details, encompassing references (title, abstracts, date of publishing, and authors), application, physical asset, level of integration, technology readiness level (TRL), input requirements, outcomes, and main capabilities (simulation, monitoring, and user interface), were systematically compiled in an online spreadsheet accessible to all authors.
5. Data analysis and results: The initial phase involved a straightforward descriptive assessment of each paper, presented in tabular format, combined with statistical analysis.
6. Results interpretation: Based on the available scientific evidence, conclusions were drawn within the context of the aforementioned primary RQs.

### 3.2. Literature Search

Scopus, Google Scholar, Taylor and Francis, MDPI, and IEEE Xplorer were utilized as search engines to explore publications related to the present investigated topic. Keyword combinations were employed of “Digital Twin” AND “agriculture”, “Digital twin” AND “livestock”, and “Digital twin” AND “forestry”. The search was extended to scrutinize references in each article for additional studies. This iterative process continued until no further relevant studies were found, concluding on 20 December 2023. Subsequently, titles and abstracts of the resulting papers were reviewed, followed by a thorough examination of the full text for relevance.

The selection criteria included were: (a) Application domain is open-field agriculture, livestock farming, or forestry; (b) Inclusion of only papers in journals and conferences, emphasizing the advantages of combining reliability and peer-reviewed validation with diverse perspectives and recent innovations in the field. Non-English studies, as well as dissertations and grey literature, were excluded to ensure that the research findings could be readily understood by a broader audience and to ensure a higher standard of reliability. This aligns with the intention of the systematic review to analyze robust and well-established evidence from reputable scholarly sources. Finally, older review papers solely focused on livestock farming [49,50], forestry [51], controlled-environment agriculture [52,53], broader agricultural purposes [54,55,56,57,58,59], or other industries, such as [60,61,62,63], were excluded. Nevertheless, they were examined for supplementary references that were not initially identified, and they also served as valuable sources of information to address various aspects related mainly to RQ4.

**Figure 1:**
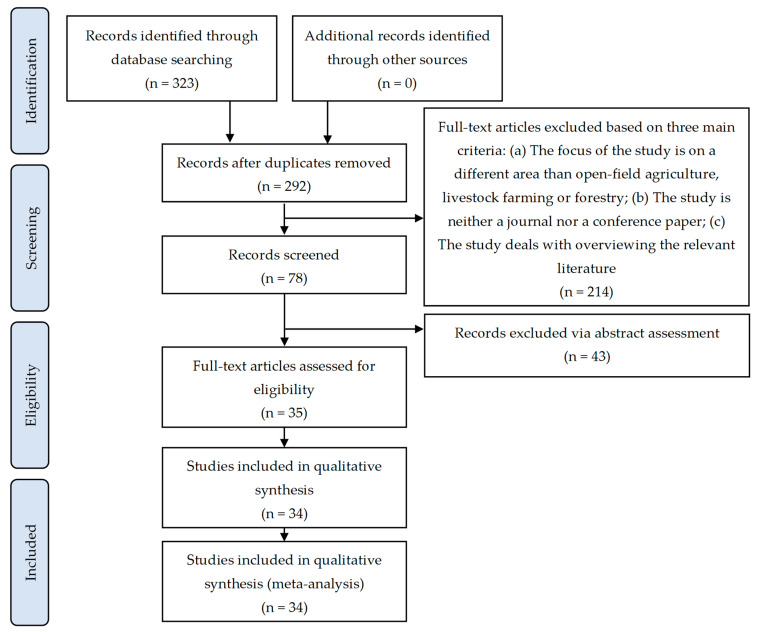
Flowchart depicting the current systematic review process for the selection of pertinent studies.

A concluding consensus meeting among the co-authors took place to deliberate on the content and suitability of the chosen papers in accordance with the above inclusion criteria and to address any divergent viewpoints. Conforming to PRISMA guidelines [48] to provide a clear representation of the methodology employed for screening the relevant literature, the systematic review identified 34 pertinent studies. The associated flowchart outlining the review process is presented in Figure 1. 

### 3.3. Level of Digital Twin Integration

The level of integration of a DT denotes the extent to which it is linked and synchronized with its physical counterpart and the broader operational environment. This integration spectrum spans from basic to advanced levels, reflecting the sophistication and efficiency of the DT system. At its development stage, there may be a one-way transfer of data from the physical entity to the DT. As integration advances, a bidirectional exchange of information can be established, allowing the DT to actively interact with and influence the physical system in real-time. A heightened level of integration enhances the DT’s capacity to offer precise real-time insights, support predictive modeling, and support more informed decision-making processes across various domains, including agriculture and forestry.

In the present study, the proposed classification of Kritzinger et al. [64] is used, which was developed on the basis of the achievable level of data integration between the physical asset and its virtual counterpart. Specifically, in exploring the integration of DT, it is imperative to delve into the distinctions among the terms “Digital Model” (DM), “Digital Shadow” (DS), and “Digital Twin” (DT). Commonly identified as digital counterparts of physical entities, these terms are frequently employed interchangeably. Nevertheless, disparities arise in the degree of data integration between the physical and digital parts. In more detail:

- A DM represents an existing or planned physical object without automated data exchange, relying on manual data integration for development. It can be utilized for various purposes like simulation and mathematical modeling. A modification in the condition or status of the physical entity does not directly impact the digital representation, and conversely, alterations in the digital object do not immediately affect the state of the physical object.
- A DS, building on the DM concept, involves automated one-way data flow from the physical to the digital object. Changes in the physical object’s state impact the digital object, but not vice versa.
- A DT, taking integration further, entails bidirectional, fully integrated data flows between a physical and digital object. In this comprehensive combination, the digital object may function as a controlling instance for the physical asset, and changes in one of them directly affect the state of the other. Other physical or digital objects may also induce changes in the DT, illustrating a dynamic and interactive relationship between the physical and digital realms.

**Figure 2:**
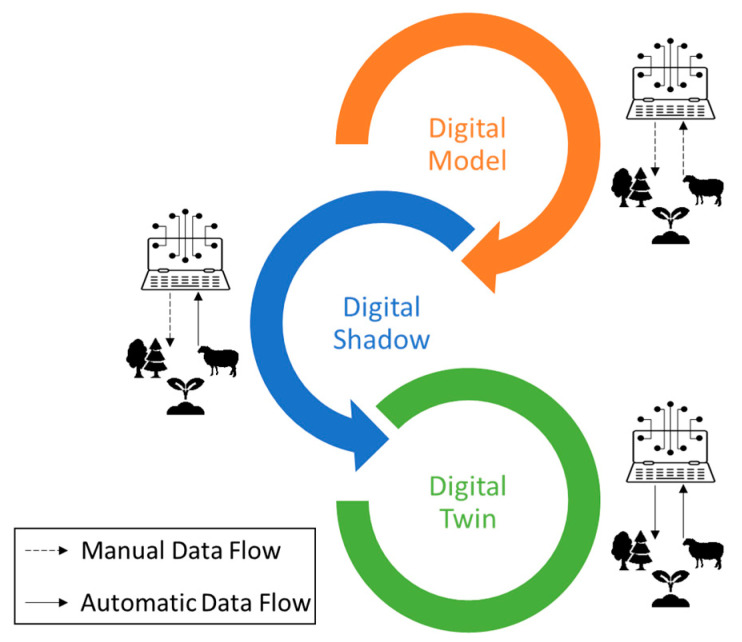
Data exchange in Digital Models, Digital Shadows, and Digital Twins.

Based on the previously outlined definitions of digital representations and the degree of interaction within cyber-physical systems, Figure 2 is introduced.

As indicative examples in the context of open-field agriculture, mirroring the above classification of digital representation, a DM can act as a detailed representation of the planned cultivation area, incorporating manual data integration for instant insights into soil, crop status, and weather conditions. Progressing from this, a DS integrates real-time sensor data, providing farmers with current condition monitoring to be further evaluated for decision-making in areas like irrigation, fertilization, etc. However, modifications in the DS do not directly affect the physical field. Advancing to the highest level of integration, a DT enables bidirectional data exchange, empowering farmers to receive real-time data and exercise remote control. This dynamic relationship between the physical field and its digital replica enhances decision-making and adaptability in the dynamic realm of agriculture.

### 3.4. Maturity Level Assessment

**Figure 3:**
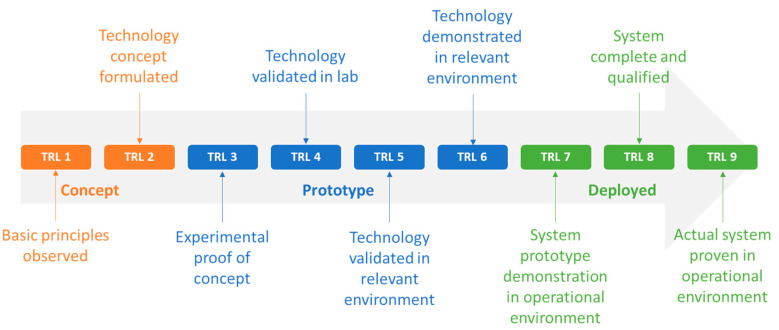
The categorization of Technology Readiness Levels (TRL) within the European Union [65] is organized into three overarching maturity levels: Concept (TRL 1–2), Prototype (TRL 3–6), and Deployed (TRL 7–9).

Overall, the TRL framework offers a standardized approach to assess technology maturity, fostering a common language among stakeholders. Additionally, TRL facilitates early risk identification and problem resolution, minimizing potential issues in later stages while fostering the successful transition of innovations from the laboratory to real-world implementation. In line with the European Union’s TRL scale [60], which is a numerical scale ranging from 1 to 9, we have categorized TRL into three main consolidated levels. In particular, the first consolidated level denotes a DT in a conceptual phase (Concept), the second involves a DT with a functional prototype (Prototype), and the third encompasses mature DT deployments in operational settings (Deployed). By employing this structured approach, summarized in Figure 3, the stage of technology development can be evaluated, informing our exploration of RQ3 and contributing to a better understanding of its applicability and potential challenges and future perspectives related to RQ4.

## 4. Results

### 4.1. Classification of the Reviewed Studies in Terms of Their Key Aspects

As highlighted above, 34 relative papers were selected based on the established criteria. Among these, 18 were published in international journals, whereas 16 were presented in international conferences. These studies are outlined in Table 1 in chronological order, whose columns summarize some key aspects related to their content.

In particular, the columns of Table 1 encompass the following key aspects:

- Type of the paper (Journal or Conference);
- The application domain (Open-field agriculture, Livestock, or Forestry);
- The physical asset that is virtually represented;
- The integration level (DM, DS, or DT), as analyzed in Section 3.3;
- The maturity level (Concept, Prototype, or Deployed), as elaborated in Section 3.4;
- The means of data acquisition;
- Input features;
- Output features;
- Main capabilities (Monitoring, Simulation, User interface).

### 4.2. Investigation of the Application Field, Level of Digital Twin Integration, and Level of Technological Maturity

**Figure 4:**
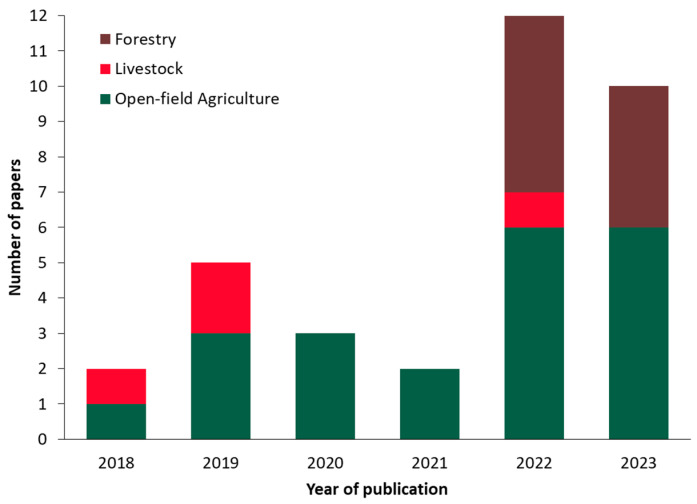
Chronological distribution of the reviewed papers according to the field of application.

Figure 4 displays a bar chart detailing the temporal distribution of the identified papers from 2018 to 2023. In particular, in 2018, only two papers were found, with one focusing on open-field agriculture and the other on livestock. The number of papers increased to five in 2019, with three in open-field agriculture and two in livestock. In 2020, all three papers focused on open-field agriculture, while in 2021, two papers were identified as exclusively related again to this domain. The year 2022 witnessed a substantial rise in the number of publications, with 12 papers, including six in open-field agriculture, one in livestock, and five in forestry. This trend continued in 2023, with ten papers: six in open-field agriculture and four in forestry. Overall, Figure 4 reflects the growing interest in DT within agriculture and forestry over the past six years, coinciding with the sector-specific expansion of Industry 4.0. However, the number of pertinent papers is relatively low since implementing DT systems in agriculture and forestry faces several challenges. The intricacy of the sectors’ operations, the reliance on dynamic environmental factors, and variability in crop, livestock, and forestry conditions pose significant challenges. Additionally, resource constraints in rural areas, seasonal variations, and issues related to data accuracy and adoption barriers further complicate the development and implementation of DT systems in these sectors. In particular, the systems to be described in a DT are dynamic, changing their properties on a seasonal basis, and are affected by several parameters that are difficult or complex to measure, model, and predict, such as the weather conditions and the soil properties, among many others. Furthermore, many of the data sources are based on indirect measurement methods or are expensive and laborious, thus limiting the number of acquired data and, hence, the accuracy.

**Figure 5:**
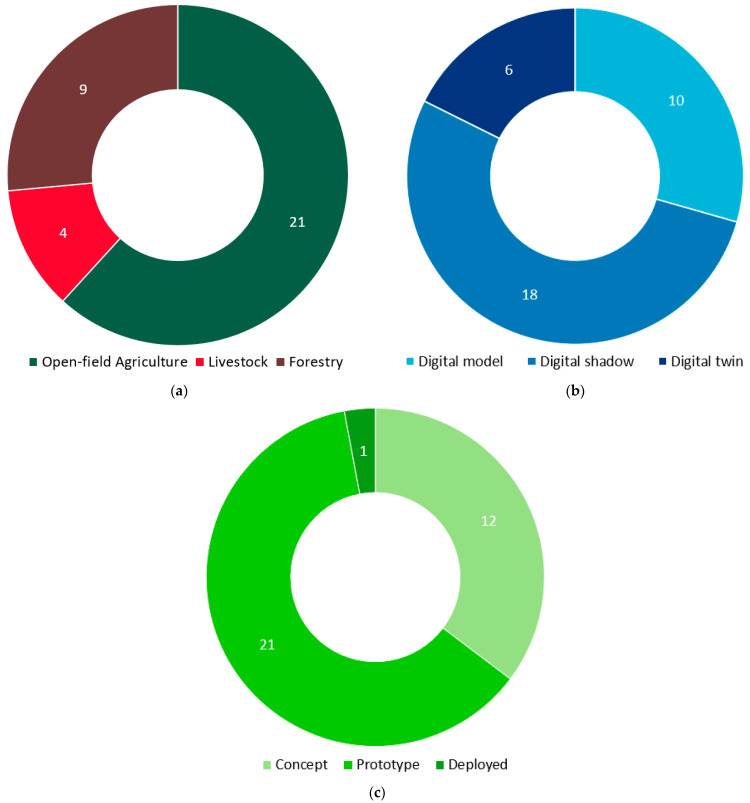
Distribution of the reviewed papers by: (**a**) Application field, (**b**) Level of integration, and (**c**) Level of technological maturity.

The majority of the selected studies are on open-field agriculture, followed by forestry and livestock, comprising 21, 9, and 4 papers, respectively, as can be depicted in Figure 5a. Notably, forestry-related DT applications were published solely in the last two years (2022, 2023), indicating a recent trend in this domain. This fact showcases a symbiotic relationship among the three examined sectors, as knowledge and insights gained from one domain often find fertile ground and relevance in the others. This interconnectivity emphasizes the importance of the present cross-disciplinary approach, as advancements in DT in one sector have the potential to affect the progress in the other sectors.

Regarding the digital representation achieved in the selected studies, only 6 of them correspond to fully integrated DTs, based on the categorization described above, attaining the necessary attributes of adequately modeled actions based on automated real-time data monitoring along with responsive feedback mechanisms. On the opposite end of the integration level spectrum, ten studies fall into the category of DMs. Given the early stage of DT implementation in the reviewed sectors, the process of modeling these complex systems emerges as a crucial prerequisite for achieving fully realized DTs. Finally, 18 studies were classified as DSs, which usually allow for real-time monitoring but do not provide bidirectional automated data flow. DSs, while not as complete as the DTs, offer insightful representations that help to understand ecosystem behavior, identify patterns, and improve models. They also allow for incremental implementation and refinement of DTs, making the journey to full implementation of digital replicas more manageable. In fact, DSs play a crucial role in bridging the divide between static models and dynamic twins, contributing significantly to the evolutionary advancement of DT technologies. The aforementioned distribution of reviewed papers by level of integration is illustrated in Figure 5b.

In the direction of getting an insight into the degree of technology maturity of the examined cyber-physical systems, the methodology analyzed in Section 3.3 was followed through the classification into “concept” (TRL 1–2), “prototype” (TRL 3–6), and “deployed” (TRL 7–9) according to the TRL scale of [65]. It is important to note that a technology is considered “deployed” once it has been demonstrated in an operational environment. If it has been validated or demonstrated in a relevant environment, it is still at the prototype maturity level. As can be deduced from Figure 5c, only one of the reviewed technologies was classified as deployed, which was presented in [74]. In the specific study, a ready-to-use cyber-physical system simulating real-time rice crop conditions was presented. The system employs an ontology-based knowledge base for plant growth in conjunction with real-time data from rice fields, enabling adaptive resource scheduling and optimal decision-making for rice cultivation. In contrast, 12 reviewed technologies were at a conceptual maturity level, signifying that they were still in the early stages of development. The majority of the technologies were at an intermediate maturity level (TRL3 to TRL6), totaling 21 instances. This indicates that these technologies have advanced beyond the conceptual stage and may have been demonstrated or validated in laboratories or relevant environments. The progression to this level suggests a notable transition from theoretical development to practical implementation. Nevertheless, the limited number of technologies at the highest maturity levels highlights the challenges associated with achieving full-scale deployment and widespread adoption in real-world settings. This can be attributed to the inherent complexity of designing and implementing DT in the agricultural and forestry context, where factors like diverse ecosystems, variable conditions, and operational intricacies contribute to a slower progression from concept to practical deployment.

### 4.3. Outline of the Main Technological Capabilities

**Figure 6:**
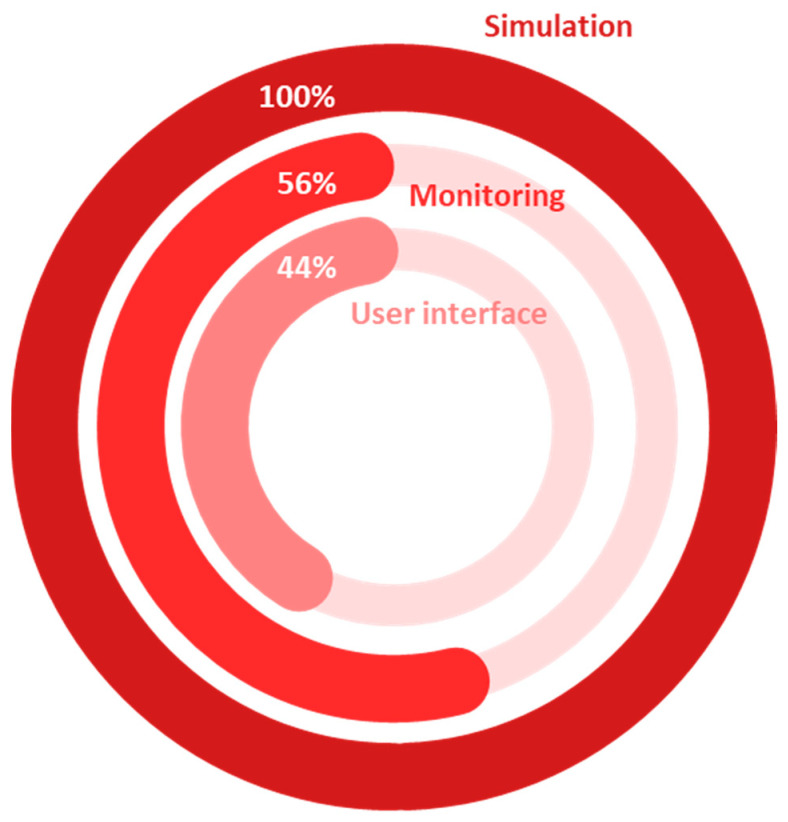
Distribution of the reviewed papers by technological capabilities.

As can be seen in Figure 6, all the reviewed studies employed simulations, with a predominant reliance on ML algorithms, occasionally supplemented by physics-based modeling. The integration of ML algorithms in agricultural and forestry-related cyber-physical systems adds substantial value by enabling data-driven decision-making and predictive analytics. ML enables the thorough analysis of complex datasets and the identification of patterns, thereby enhancing the efficiency and accuracy of these, leading to enhanced resource management and optimized results [22,23]. Furthermore, nearly half of the studies (56%) incorporated real-time monitoring and user interfaces (44%), indicative of an advanced level of integration, aligning with the progression towards DSs and DTs. This signifies a maturation of technologies in the direction of more sophisticated and interactive cyber-physical systems capable of real-time data analysis and decision support. The incorporation of real-time monitoring and user interfaces also enhances the practical utility of these systems, fostering more informed and timely decision-making in open-field agriculture, livestock farming, and forestry.

### 4.4. Summary of the Main Physical Assets, Data Acquisition Methods, and Input and Output Features

**Figure 7:**
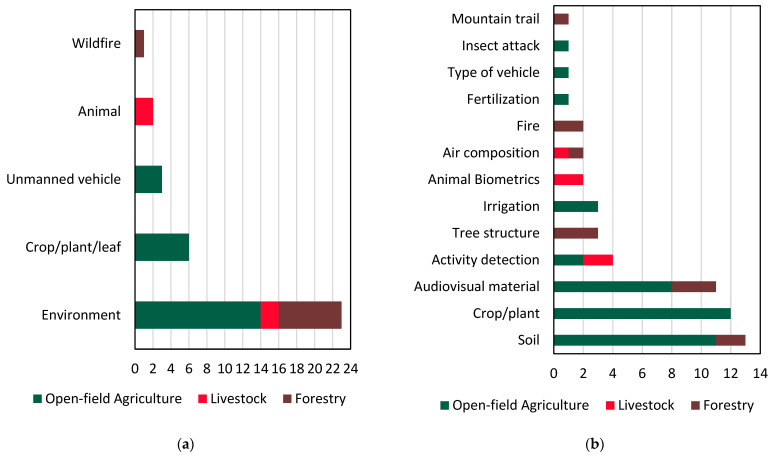

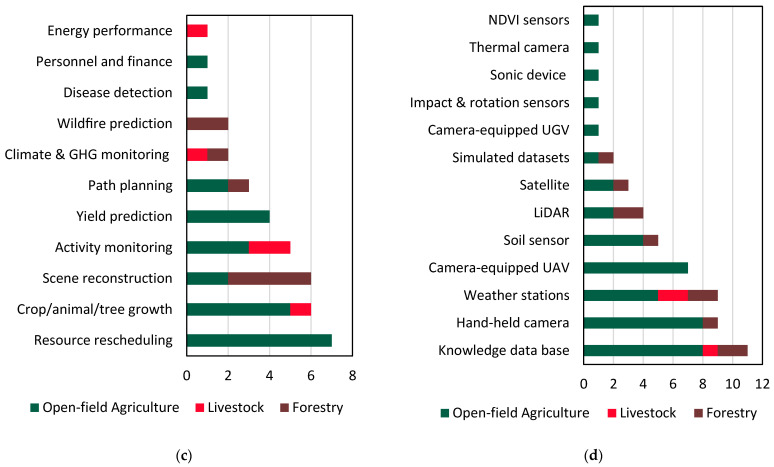
Frequently employed elements: (**a**) Physical assets, (**b**) Input features, (**c**) Output features, and (**d**) Means of data acquisition in relation to the number of papers across application domains.

As depicted in Figure 7a, the majority of the reviewed studies dealt with developing digital replicas of the surrounding environments, usually the cultivated fields (e.g., orchards, vineyards, rice fields, etc.), animal farms, and forests, and more rarely, the investigated crop, plant, or the animal itself. Finally, three studies were concerned with the modeling of ground and aerial vehicles, while one study modeled the spread of fire within a forest.

Figure 7b sheds light on the diverse input features frequently integrated into the studies under review, offering crucial information for modeling or monitoring. Notably, soil data emerges as an essential input, emphasizing its significance in shaping DTs for open-field agriculture and forestry contexts. Crop/plant characteristics, another prominent input, emphasize the importance of plant-related parameters. Additionally, audiovisual material is commonly used, contributing to a holistic understanding of the surrounding environment. Other input characteristics, such as activity detection, tree structure, irrigation, animal biometrics, air composition, fire, fertilization, type of vehicle, insect attack, and mountain trail, showcase the broad spectrum of elements considered in the reviewed studies.

Figure 7c illustrates the prevalent output features derived from DTs across the reviewed studies, capturing the outcomes and insights generated by these systems. Resource rescheduling arises as a prominent output, reflecting the focus on optimizing resource allocation and enhancing operational efficiency. The emphasis on monitoring and predicting growth, whether in crops, animals, or trees, underscores the central character of DTs in agriculture and forestry management. Scene reconstruction features are also prominent, indicating the interest in creating detailed and accurate digital replicas of the physical environment. Activity monitoring, yield prediction, and path planning highlight the multifaceted capabilities of DTs in providing valuable insights for decision-making. Moreover, the incorporation of features, such as climate and greenhouse gas (GHG) monitoring, wildfire prediction, disease detection, and personnel and finance management, demonstrates the extensive range of applications for DTs. These applications are instrumental in addressing a variety of challenges within the agricultural and forestry domains to cater to the complex and dynamic nature of these ecosystems. Interestingly, energy performance is illustrated as one of the least addressed elements. This is bound to change in the future, given the global efforts on climate change and the pursuit of net zero economies. As such, many DT applications could be complemented with aspects relating to renewable energy production and energy efficiency [100,101].

Finally, as can be deduced from Figure 7d, a variety of IoT sensors have been used in all examined application fields, while remote sensing has been utilized in many studies associated mainly with open-field agriculture, either utilizing datasets acquired by UAVs equipped with cameras or using satellite data. This result agrees with the findings of the recent review on ML in agriculture [23], which highlighted the increasing usage of UAVs versus satellites. On-site camera photography has also been employed in various studies, while Moysiadis et al. [79] used a camera-equipped UGV, highlighting their role in capturing visual data, usually to be utilized as input in Deep Learning models [102]. A thermal camera has also been utilized in one of the studies to monitor the physiological changes of bananas throughout storage [78]. LiDAR technology, with its capability for detailed terrain mapping, appears in a significant number of studies, providing a specialized tool for precise data collection.

Weather stations are also instrumental in collecting essential environmental data for DTs. In open-field agriculture, weather data are vital for monitoring growing conditions, assessing risks related to weather events or other biological factors such as the occurrence of pests and diseases, and optimizing irrigation or crop protection schedules. Livestock farming benefits from measuring environmental parameters, including NH_3_ and CO_2_ levels, to manage animal welfare, especially during extreme conditions. In forestry, environmental data are essential for fire risk assessment and understanding the ecosystem dynamics. Soil sensors are also crucial primarily in open-field agriculture for monitoring key soil parameters, such as temperature, moisture content, nutrient status, salinity, and pH, contributing to precision agriculture practices.

Interestingly, the utilization of various knowledge databases is the main source of data acquisition, showcasing their relevance and contributing valuable insights. These databases encompass a wide array of information sources, including historical records, experimental data, ground-based sensor readings, remote sensing imagery, and expert knowledge. By leveraging these diverse sources, DTs can access comprehensive information about environmental conditions, crop health, animal behavior, forest dynamics, and other relevant parameters. Simulated datasets have also been used [68,99] to enhance DTs by providing controlled training environments, filling data gaps, and enabling learning from hypothetical scenarios. Sonic devices were utilized in one study for the detection of animal intrusion [80], while NDVI (Normalized Difference Vegetation Index) sensors in [82] provided crucial insights into crop health. Finally, the utilization of impact and rotation sensors was described in [67] for simulating the impacts the potatoes face during harvest. This “digital potato” was suggested to be used as a digital twin of real potatoes to aid in adjusting the harvester correctly, minimizing the damages to the harvested crop.

## 5. Discussion

This section is organized into four distinct subsections, each aligned with a specific RQ to systematically analyze key facets of DT in the context of open-field agriculture, livestock farming, and forestry. The focus of Section 5.1 is on unraveling how the DTs are conceptualized and defined in the above related systems. Section 5.2 discusses the current applications of DTs in these domains, thus, shedding light on the various ways in which DTs are employed. Section 5.3 delves into the extent of achieved integration level of DTs, providing insights into the obstacles and advancements encountered in the process. Finally, Section 5.4 tackles the exploration of open questions, challenges, and future perspectives, offering a comprehensive overview of the critical considerations and potential advancements in the landscape of DT in the reviewed sectors.

### 5.1. RQ1: How Are DTs Conceptualized and Defined in Agricultural and Forestry Systems?

VanDerHorn and Mahadevan [61] identified a gap in the existence of a clear definition of the DT concept, emphasizing the need for practical examples of DT implementation. After reviewing 46 different definitions describing the DTs, they proposed the following as the most appropriate general description: “*a virtual representation of a physical system (and its associated environment and processes) that is updated through the exchange of information between the physical and virtual systems*”. This general definition adequately describes the DTs in closed production and operational environments met mainly in an industry where the systems are finite and there are distinct boundaries of the operations and sub-systems.

However, due to the open nature of agricultural and forestry environments, the definition of DTs cannot clearly reflect the dynamic and unpredictable conditions that are met in these environments. This was the main reason for the delay in the development and implementation of DTs in these sectors [103]. In the current review study, a detailed description of the DTs and the categorization of the different levels of implementation in agriculture and forestry were discussed. The review resulted in 34 papers describing DT applications in agriculture and forestry, most of which were at conceptual and prototyping levels. Based on the literature analysis, these studies included all levels of implementation: DM, DS, and DT. This depicts the researchers’ conceptualization of DT in agriculture and forestry. Given the current state of the art, along with the intricate nature of the described systems in these domains, DMs and DSs were considered as DTs. It should be stressed that according to most definitions, these are essential elements of fully deployed DTs, which also require two-way data exchange between the physical and the digital twin. However, due to the high complexity of the biotic and abiotic systems involved, in some cases, the maximum level of implementation can only reach the DS level.

Given the aforementioned discussion and the fact that DTs are rapidly evolving, there is a need to export a clear definition of DTs in the reviewed sectors for the first time, to the best of the authors’ knowledge. To this end, the above general definition provided in [96] can be adjusted to reflect the current needs in open-field agriculture, livestock farming, and forestry as follows:

*“Digital twin in the unpredictable environments of agriculture and forestry can be defined as a virtual representation of a physical system, dynamic or static, and its associated environment and processes, both biotic and abiotic, that at full implementation may be updated through the exchange of information between the physical and virtual systems”.*

In the augmented definition, the phrase “*in the unpredictable environments of agriculture and forestry*” was added to emphasize the unpredictable nature of agriculture and forestry environments. The term “*dynamic or static*” was added to reflect both the dynamic nature of living organisms (flora and fauna) and the static systems and machinery to be digitally twinned. Furthermore, the corresponding environment and processes are divided into “*biotic and abiotic*” to highlight the complex nature of the coexistence of living and non-living system entities. Finally, the phrase “*at full implementation may be*” was added to underscore the current limitations in achieving fully developed DTs in complex biotic and abiotic environments.

### 5.2. RQ2: What Are the Current Applications of DTs in Open-Field Agriculture, Livestock, and Forestry?

The present review reveals a growing interest in DTs within agriculture and forestry, marked by a notable increase in relevant literature in the last two years (i.e., 2022 and 2023). While the number of papers is relatively low compared to the potential of employing DT in these sectors, the trend aligns with the sector-specific expansion of Industry 4.0. Open-field agriculture draws most of the attention, followed by forestry and livestock. A recent surge in forest-related DT applications indicates a growing tendency.

DTs play a fundamental role in crop monitoring, offering real-time insights into crop conditions through the integration of data from diverse sources such as inter alia, camera-equipped UAVs [70,74,75,77,85,86] and UGVs [79], satellites [77,83], soil sensors [70,75,81,83], weather stations [74,75,81,82,83], and knowledge databases [66,71,72,73,74,77,82,84]. This integration allows for comprehensive analyses, aiding farmers in optimizing critical open-field agricultural practices like irrigation, fertilization, and pest control. In addition, DTs empower farmers with simulation modeling capabilities, enabling them to envision and assess various what-if scenarios. By simulating the potential impact of factors, such as weather patterns, soil conditions, and resource management strategies, farmers can make informed decisions to enhance crop growth and yield. In summary, the multifaceted capabilities of DTs indicatively include resource rescheduling [72,73,74], growth monitoring and prediction [73,74,82,84], yield prediction [83,84,85], scene reconstruction [69], path planning [86], and addressing challenges like disease detection [70] and personnel and finance management [74].

DTs have also become invaluable tools in livestock management, offering a multifaceted approach to enhance various aspects of livestock farming. By creating virtual representations of individual animals and entire animal farms, DTs enable personalized nutritional optimization, improving overall health and productivity. Reproductive management benefits from DTs by predicting optimal breeding times. The examination of animal behavior via integrated IoT sensors aids in identifying early signs of illness, facilitating timely intervention for improved animal welfare. Disease detection and prevention are heightened by improved continuous health monitoring, minimizing the spread of illnesses within the herd. Environmental factors are usually taken into account with DTs, optimizing conditions in stables or pastures [87]. Precision livestock farming is achieved by optimizing resource use, while wearable technology integration supports real-time monitoring of individual animals [88,90]. Predictive modeling based on real-time and historical data offers farmers data-driven insights for informed decision-making in livestock management.

DTs also contribute to the advancement of sustainable forestry practices. In particular, through scene reconstruction [91,97], DTs offer valuable insights for resource management and environmental monitoring. By leveraging data from various sources, including LiDAR [91,93] and remote sensing technologies [95], DTs can create three-dimensional virtual representations of forest landscapes. These virtual reconstructions offer in-depth insights into terrain and canopy cover, assisting foresters in accurate spatial assessments and the identification of potential logging sites while also enabling efficient programming for infrastructure placement. Tree segmentation and species recognition are also important tasks in forest inventory activities, enabled by the DT applications, which provide valuable insight for optimal forest management. The detailed information provided by virtual reconstructions enables also foresters to optimize path planning for UAV within the forestry landscape through the utilization of DTs [92]. Moreover, DTs contribute to wildfire prediction by analyzing environmental factors and monitoring changes in real-time, enhancing early detection capabilities as well as GHG monitoring, thus, aiding in the assessment climate change mitigation. Nevertheless, there is still an observed limitation in DTs in terms of addressing climate mitigation activities of agriculture and forestry, which play a crucial role in the energy transition. Current work in the field, such as [104], could be mirrored in DTs.

Overall, as DTs continue to evolve, their diverse applications hold promise for revolutionizing agricultural and forestry practices in the pursuit of more informed, resilient, and sustainable approaches.

### 5.3. RQ3: To What Extent has DT Integration Been Achieved?

**Figure 8:**
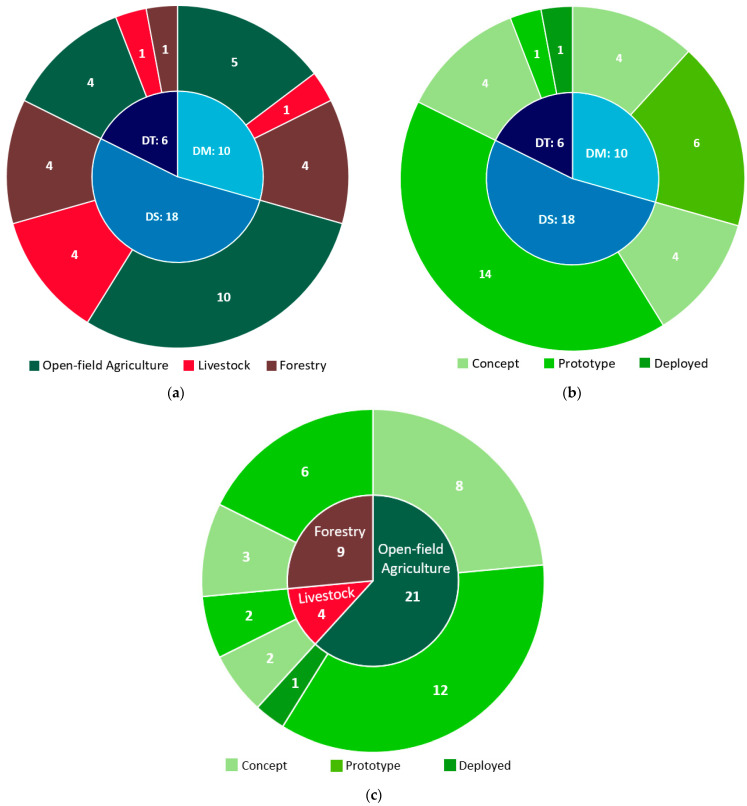
A consolidated illustration of the reviewed papers, exploring the distribution of the levels of integration in conjunction with (**a**) The application field, (**b**) The level of technological maturity, and (**c**) The distribution of the level of technological maturity for each application field.

Figure 8a–c presents a comprehensive overview of the reviewed papers, examining the distribution of integration levels alongside the application field and technological maturity. In Figure 8a, it is evident that most studies in open-field agriculture are situated at the DS level, followed by DM and DT. Similarly, studies focusing on livestock predominantly fall into the DS category. In the context of forestry systems, the primary classifications are both DMs and DSs. This distribution reflects the varying degrees of technological advancement and application maturity within each sector, emphasizing the need for tailored approaches and advancements in technology to achieve higher levels of integration. As depicted in Figure 8b, only one study was classified at the deployed level (represented by dark green), which was categorized as DT, indicative of its practical implementation [74]. The DSs and DMs, situated mainly at the prototype level, highlight their intermediary state of development, thereby demonstrating progress but not yet at the fully deployed stage. Finally, Figure 8c illustrates a breakdown of the level of technological maturity across different application fields. Open-field agriculture-related applications show a significant presence at the prototype level, indicating remarkable development in this area, followed by application maturity at the concept level. Livestock-related applications exhibit a smaller number of studies overall, with a balanced representation between concept and prototype levels. Forestry-related applications also demonstrate a mix of concept and prototype-level studies, reflecting ongoing technological advancement in this field.

Overall, the integration of DTs in the reviewed sectors has made remarkable strides in recent years, underlining the growing recognition of their transformative potential. The increasing utilization of real-time data from diverse sources reflects a considerable degree of DT integration. Nevertheless, despite the remarkable progress mentioned above, it should be mentioned that DTs are still at an early or intermediate stage of development. Achieving full integration of DTs poses challenges, with nearly half of the studies reviewed operating at the DS stage. On the one hand, this highlights the complexity of realizing complete integration. On the other hand, DSs offer valuable insights into the development process of DTs for new applications. In addition, approximately one-third of the examined studies were classified as DMs. Given that DTs are in their infancy in agriculture and forestry, developing models for these systems can be intricate. Notably, in the realm of digital twinning, creating DMs is a necessary precursor, a factor that requires consideration when evaluating DTs.

Stakeholders can use the above stages of DM and DS to deepen their understanding of entities, paving the way for exploring and evaluating approaches to full integration. Utilizing DMs and DSs as frameworks for testing new technologies and models enables incremental progress. The exact level of automation or required interaction with the real-world entity remains somewhat unclear, emphasizing the need to differentiate between DMs, DSs, and DT. This is an aspect often overlooked in the existing literature on reviews of DT integration.

### 5.4. RQ4: “What Are the Open Questions, Challenges, and Future Perspectives?”

While significant steps have been made in advancing DTs within open-field agriculture, livestock farming, and forestry, a variety of unresolved issues persist. In particular, the very dynamic nature of these ecosystems presents unique challenges for DTs to accurately replicate dynamic behavior, surpassing requirements found in many other sectors. Furthermore, agricultural products, being living entities, inherently exhibit diversity and complex behavior.

Another particular characteristic of these ecosystems is that the digital representation of them is not a singular DT concern; rather, it involves a wide range of interconnected objects at different levels of granularity, introducing object complexity. These primary objects encompass:

- Inputs like feed, fertilizers, and pesticides;
- Entities associated with production, such as growing crops, trees, or animals and resources like agricultural fields, livestock farms, forests, personnel, tractors, drones, and other machinery;
- Outputs, including harvested crops, cut trees, and milk, for example.

The granularity of a DT refers to the level of detail or precision at which the digital representation mirrors its physical counterpart. In other words, it describes how finely the DT models represent the real-world object or system, ranging from coarse representations to highly detailed, individualized models. Choosing the appropriate granularity level is essential to maximize the efficiency and effectiveness of a DT, striking a balance between excessive detail, which can be computationally demanding and slow, and insufficient detail, which may not offer adequate insights for decision-making [53,105].

Detailed DT solutions also demand the regular storage and processing of substantial volumes of data for the continuous creation and updating of digital models. The current DTs in the reviewed domains encounter common challenges alongside many elements comprising ICT. These include issues related to data management, standardization, security, and privacy. Addressing ethical considerations is also imperative as the routine collection of sensitive data becomes widespread. The question of data ownership becomes particularly crucial in applications utilizing uniquely identifiable information. While technological and legal solutions can play a role in resolving these concerns, it is essential to directly tackle these issues through establishing agreements with and among relevant stakeholders [55]. Additionally, DT advancement necessitates farmers, livestock owners, and forestry managers to enhance their data handling capabilities to cope effectively with such extensive datasets. For this purpose, investment in IoT sensors, software, and infrastructure development is essential in conjunction with comprehensive training to ensure that stakeholders grasp the intricacies of the complex technology and apply it correctly.

The scarcity of practical and expansive datasets directly obtained from fields emphasizes the need for comprehensive, field-specific information. Advancing this technology requires more efficient digital models and scalable computational architectures for rapid information processing. Overcoming challenges related to the integration of diverse data types, such as audio recordings, images, videos, and databases with data coming from in situ measurements, inter alia, remains a challenging task.

Looking forward, exploring the potential of emerging technologies, like cloud computing, extended reality, and 5G networks, is essential for enhancing the capabilities of DTs in effectively capturing, analyzing, and responding to dynamic environmental conditions. Advancements in AI, such as large language models like ChatGPT, can considerably aid the development of DTs in agriculture and forestry, offering robust capabilities for decision-making processes by analyzing and interpreting complex datasets. However, it is crucial to assess the necessity and resource efficiency of such advanced AI technologies against simpler paradigms, which may provide sufficient functionality for a number of tasks, thus optimizing both computational and energy resources. At the same time, it has to be highlighted that it is also imperative to prioritize the development of user-friendly ICT, including open-source platforms to optimize processes and accompanying costs, accessible user interfaces of comfortable control, and smartphone applications to create value-added services and facilitate widespread comprehension of DT insights among non-expert stakeholders.

## 6. Conclusions

The present review focuses on the increasing interest in adopting DTs in open-field agriculture, livestock farming, and forestry. Despite inherent challenges such as the complexity of the sectors, dynamic environmental factors, and possible adoption barriers, these sectors exhibit noteworthy interconnectedness and mutual relevance. In brief, over half of the reviewed studies deal with open-field agriculture, followed by forestry and livestock farming in descending order of prevalence. Only a limited number of studies feature fully integrated DTs, whereas the majority are at an intermediate maturity level, indicating progress beyond the conceptual stage. Moreover, all studies leverage simulations, mainly via ML algorithms, contributing to data-driven decision-making, while nearly half of them incorporate real-time monitoring and user interfaces. The variety of the investigated physical assets, as well as the diverse means of data acquisition and input/output features, highlights the complexity of these cyber-physical systems, addressing the intricate nature of agricultural and forestry ecosystems. This study also aimed to shed light on the unresolved queries, obstacles, and future prospects, providing a thorough overview of key factors and potential advancements in the realm of DTs within the examined sectors.

In a nutshell, full implementation of DTs describing all the biotic and abiotic systems in agricultural and forest environments proves to be a major challenge within the realm of DTs, accompanied by the need for accessible, real-time, and high-quality data, while ethical considerations, especially concerning data privacy, should be prioritized. This review aimed to provide a detailed insight into the status and deployment level of the state-of-the-art studies in the agriculture and forestry sectors, emphasizing the nature-imposed limitations of deploying fully developed DTs. Based on these limitations, a new definition is proposed that is tailored to the specific attributes of complex biotic and abiotic systems.

Finally, beyond rapidly testing hypotheses or evaluating the outcomes of decisions, the DT framework has the potential to significantly broaden farmers’ knowledge. While the models expand, incorporating data on factors such as soil composition, weather, and animal physiology, they will also draw on the collective expertise of farmers and other relevant contributors. Therefore, an important future perspective involves fostering the development of efficient techniques by integrating expert knowledge from diverse stakeholders in computing science, agriculture, forestry, and the private sector. This collaborative and multi-disciplinary approach will contribute to designing realistic solutions intricately connected with decision-making processes, transcending beyond isolated efforts.

## Figures and Tables

**Table 1:** Compilation of the reviewed papers, classified by category and year, along with some key aspects.

Ref	Type	Application	Physical Asset	Integration Level	Maturity Level	Means of Data Acquisition	Input	Output	Capabilities
Villani et al. (2018) [66]	Conf	Open-field agriculture	Vineyard	DM	Proto-type	Knowledge data base	Soil, crop, weather, and water table data	Crop irrigation needs	Simulation
Kampker (2019) et al. [67]	Conf	Open-field agriculture	Plastic Potato	DS	Proto-type	Impact and rotation sensors	Shocks and blows as detection; rotation speed	Harvest optimization	Monitoring; simulation; user interface
Tsolakis et al. (2019) [68]	Journal	Open-field agriculture	Orchard, UGV	DS	Proto-type	Emulation tool; LiDAR; Hand-held camera	Soil data; type of UGV; simulation environment	UGV navigation	Monitoring; simulation; user interface
Moghadam et al. (2019) [69]	Journal	Open-field agriculture	Orchard	DS	Proto-type	LiDAR; Hand-held camera	Stress, health; fruit quality; images	Canopy structural characteristics	Monitoring; simulation
Angin et al. (2020) [70]	Journal	Open-field agriculture	Field	DS	Proto-type	Soil sensor nodes; camera-equipped UAV	Soil and crop data; images	Plant diseases and weeding detection; energy consumption	Simulation
Skobelev et al. (2020) [71]	Conf	Open-field agriculture	Wheat	DS	Concept	Knowledge data base	Soil, plant growth phases, fertilizing and weather data; insect attack	Yield impact of emerging events	Monitoring; simulation; user interface
Skobelev et al. (2020) [72]	Conf	Open-field agriculture	Plant	DS	Concept	Knowledge data base	Soil, plant growth phases and weather data	Resource rescheduling	Monitoring; simulation; user interface
van Evert et al. (2021) [73]	Conf	Open-field agriculture	(i) Grass	DM	Concept	Knowledge data base	Soil, grass, fertilization, mowing, grazing, groundwater depth, and weather data	Resource rescheduling	Simulation
			(ii) Potato	DM	Concept	Knowledge data base	Crop, fertilizing, and weather data	Crop growth	Simulation
Skobelev et al. (2021) [74]	Conf	Open-field agriculture	Rice field	DT	Deployed	Knowledge data base; weather stations; camera-equipped UAV	Soil and weather data; rice variety; images	Resource rescheduling; personnel and finance; plant growth	Monitoring; simulation; user interface
Awais et al. (2022) [75]	Journal	Open-field agriculture	Field	DT	Concept	Camera-equipped UAV; soil sensors; weather stations	Images; soil, weather, and irrigation data	Crop water status	Monitoring; simulation; user interface
Li et al. (2022) [76]	Journal	Open-field agriculture	Leaf	DM	Proto-type	Hand-held camera	RGB images	Leaf reconstruction	Simulation
Madeira et al. (2022) [77]	Conf	Open-field agriculture	Vineyard	DS	Proto-type	Knowledge database; satellite; camera-equipped UAV	Remote sensing images; vineyard-related data	Land and plant monitoring	Monitoring; simulation; user interface
Melesse et al. (2022) [78]	Conf	Open-field agriculture	Banana	DM	Concept	Thermal camera	Banana images	Fruit quality evolution	Simulation
Moysiadis et al. (2022) [79]	Journal	Open-field agriculture	Orchard	DS	Proto-type	Camera-equipped UGV	Video, images	Hand gesture-enabled HRI	Monitoring; simulation; user interface
Teschner et al. (2022) [80]	Conf	Open-field agriculture	UAV; field	DT	Concept	Beacon equipped with sonic device and hand-held camera	Acoustic signals; videos	Animal intrusion detection	Monitoring; simulation; user interface
Alves et al. (2023) [81]	Journal	Open-field agriculture	Irrigation system	DS	Proto-type	Soil probe; weather station; irrigation system	Soil, weather, crop, and irrigation data	Irrigation guidelines	Monitoring; simulation; user interface
Kalyani et al. (2023) [82]	Journal	Open-field agriculture	Arable field	DT	Concept	Weather stations; humidity and NDVI sensors; knowledge database	Soil, weather, and crop data	Crop growth prediction	Monitoring; simulation; user interface
Khatraty et al. (2023) [83]	Conf	Open-field agriculture	Rice field	DS	Proto-type	Soil sensors; weather station; hand-held cameras; satellites	Soil, weather, and crop data	Vegetation index and yield prediction	Monitoring; simulation
Skobelev et al. (2023) [84]	Conf	Open-field agriculture	Plant	DS	Prototype	Knowledge database	Soil, crop, and weather data	Plant growth and development; yield prediction	Monitoring; simulation
Zarembo et al. (2023) [85]	Conf	Open-field agriculture	Orchard	DS	Concept	Camera-equipped UAV	Fruit and flowers per tree; infected fruits	Yield forecast	Simulation
Zhao et al. (2023) [86]	Journal	Open-field agriculture	Field; tractor	DM	Prototype	Camera-equipped UAV	UAV imagery	Path planning	Simulation
Jo et al. (2018) [87]	Conf	Livestock	Pig farm	DT	Concept	Environmental sensors	CO_2_, dust, and environmental data	Optimal temperature; CO_2_	Monitoring; simulation; user interface
Erdélyi and Jánosi (2019) [88]	Journal	Livestock	Pig	DM	Concept	Knowledge data base	Pig temperature, heat transfer parameter	Feed consumption; weight growth	Simulation
Jo et al. (2019) [89]	Conf	Livestock	Pig farm	DS	Proto-type	Weather station	Weather data	Energy-related performance	Simulation
Han et al. (2022) [90]	Journal	Livestock	Cattle	DS	Proto-type	Wearable device	Animal movement and location; free grazing time	Cattle activity monitoring	Monitoring; simulation
Wang et al. (2022) [91]	Journal	Forestry	Tree	DS	Proto-type	LiDAR; in situ measurements of tree variables	Tree structure	3D tree skeleton reconstruction	Simulation
Bae et al. (2022) [92]	Conf	Forestry	Forest	DS	Proto-type	Knowledge data base	Mountain trail	UAV Flight plan	Simulation
Buonocore et al. (2022) [93]	Journal	Forestry	Forest	DT	Concept	LiDAR, soil moisture sensors; weather stations; flux towers	Soil, weather data; tree physical and physiological processes	Various forest- management-related applications	Monitoring; simulation; user interface
Sanchez-Guzman et al. (2022) [94]	Conf	Forestry	Forest	DM	Concept	Κnowledge data base	Duration of combustion; tree structure	Ignition point prediction	Simulation
Jiang et al. (2022) [95]	Journal	Forestry	Forest	DM	Proto-type	Satellite	Historical satellite images	Forest future image forecast	Simulation
Li et al. (2023) [96]	Journal	Forestry	Forest	DS	Proto-type	Hand-held camera	Tree diameter; images; videos	Tree growth	Monitoring; simulation; user interface
Liu et al. (2023) [97]	Journal	Forestry	Forest	DM	Prototype	Terrestrial laser scanner; spectrometer	Images; structural and spectral data	Forest scene reconstruction	Simulation
Silva et al. (2023) [98]	Journal	Forestry	Forest	DS	Concept	Environmental sensors; knowledge data base	GHG emissions	GHG monitoring and modeling	Monitoring; simulation; user interface
Zhong et al. (2023) [99]	Journal	Forestry	Wildfire	DM	Prototype	Simulated datasets	Weather, soil and fire data	Wildfire prediction	Simulation

Conf: Conference; DM: Digital Model; DS: Digital Shadow; DT: Digital Twin; GHG; Greenhouse gases; HRI; Human–robot Interaction; LiDAR; Light Detection and Ranging; NDVI: Normalized Difference Vegetation Index; RGB: Red Green Blue; UAV: Unmanned Aerial Vehicle; UGV: Unmanned Ground Vehicle; 3D: Three dimensional.

## Data Availability

Not applicable.
